# Effect of Carotenoid Supplemented Formula on Carotenoid Bioaccumulation in Tissues of Infant Rhesus Macaques: A Pilot Study Focused on Lutein

**DOI:** 10.3390/nu9010051

**Published:** 2017-01-10

**Authors:** Sookyoung Jeon, Martha Neuringer, Emily E. Johnson, Matthew J. Kuchan, Suzette L. Pereira, Elizabeth J. Johnson, John W. Erdman

**Affiliations:** 1 Division of Nutritional Sciences, University of Illinois at Urbana-Champaign, Urbana, IL 61801, USA; sjeon17@illinois.edu; 2 Oregon National Primate Research Center, Oregon Health and Science University, Beaverton, OR 97239, USA; neuringe@ohsu.edu (M.N.); johnsemi@ohsu.edu (E.E.J.); 3 Abbott Nutrition, Columbus, OH 43215, USA; matthew.kuchan@abbott.com (M.J.K.); suzette.pereira@abbott.com (S.L.P.); 4 Jean Mayer United States Department of Agriculture Human Nutrition Research Center on Aging, Tufts University, Boston, MA 02111, USA; Elizabeth.Johnson@tufts.edu; 5 Department of Food Science and Human Nutrition, University of Illinois at Urbana-Champaign, Urbana, IL 61801, USA

**Keywords:** carotenoids, lutein, bioaccumulation, brain, retina, Rhesus Macaques, infants, formula

## Abstract

Lutein is the predominant carotenoid in the developing primate brain and retina, and may have important functional roles. However, its bioaccumulation pattern during early development is not understood. In this pilot study, we investigated whether carotenoid supplementation of infant formula enhanced lutein tissue deposition in infant rhesus macaques. Monkeys were initially breastfed; from 1 to 3 months of age they were fed either a formula supplemented with lutein, zeaxanthin, β-carotene and lycopene, or a control formula with low levels of these carotenoids, for 4 months (*n* = 2/group). All samples were analyzed by high pressure liquid chromatography (HPLC). Final serum lutein in the supplemented group was 5 times higher than in the unsupplemented group. All brain regions examined showed a selective increase in lutein deposition in the supplemented infants. Lutein differentially accumulated across brain regions, with highest amounts in occipital cortex in both groups. β-carotene accumulated, but zeaxanthin and lycopene were undetectable in any brain region. Supplemented infants had higher lutein concentrations in peripheral retina but not in macular retina. Among adipose sites, abdominal subcutaneous adipose tissue exhibited the highest lutein level and was 3-fold higher in the supplemented infants. The supplemented formula enhanced carotenoid deposition in several other tissues. In rhesus infants, increased intake of carotenoids from formula enhanced their deposition in serum and numerous tissues and selectively increased lutein in multiple brain regions.

## 1. Introduction

Lutein, a yellow xanthophyll pigment, cannot be endogenously synthesized. Therefore, humans depend on dietary sources of this carotenoid, especially from green leafy vegetables, like kale, spinach, and broccoli [1]. Lutein and zeaxanthin are uniquely concentrated in the fovea of the primate retina and are known for their beneficial roles in eye health as antioxidants and blue light filters [2]. Intake of lutein and zeaxanthin from dietary sources is associated with a decreased risk of age-related macular degeneration (AMD), a leading cause of irreversible blindness in adults [3]. In addition to lutein’s impact on eye health, a recent meta-analysis reported that higher lutein consumption and higher lutein blood levels were associated with a lower risk of coronary heart disease, stroke, and metabolic syndrome [4]. Furthermore, lutein is increasingly being implicated as having a role in cognitive function [5]. Such a role is supported by recent studies showing that lutein was selectively deposited in the brains of infant and older adults [6,7]; that lutein supplementation was associated with improved cognitive function in older female subjects [8]; and that macular pigment optical density (MPOD) was related to cognitive function in older adults [9,10,11].

Emerging evidence suggests that lutein plays important roles in early human development. Lutein was the most abundant carotenoid in cord plasma, and its concentration was strongly correlated with maternal plasma lutein, suggesting a possible role of lutein in the neonatal period [12]. Lutein is the predominant carotenoid in brain in both infants and older adults, and its preferential accumulation in the infant brain implies a possible role in central nervous system development [5,13]. In a metabolomic study, lutein concentrations in infant brain were significantly correlated with neurotransmitters that are involved in neuronal proliferation and maturation, neurite outgrowth and synapse formation [14]. In addition, lutein administration in the first hours of life increased biological antioxidant potential and decreased oxidative stress [15]. Since breast milk or infant formula are the only dietary sources of lutein before solid food is introduced [13], it is critical to understand how dietary lutein relates to tissue bioaccumulation during early life.

Lutein is poorly absorbed and present in negligible levels in the retina and brain of most experimental animal models. Amongst non-human animals, only primates selectively accumulate lutein and zeaxanthin in their retina/macula and brain [16]. Li et al. recently suggested that this unique accumulation in primate retina is related to the inactivity of the primary lutein mammalian carotenoid cleavage enzyme, β,β-carotene 9′,10′-oxygenase (BCO2) [17] due to loss of an alternate splice site. On the other hand, other studies suggested that the activity of human BCO2 is conserved and dependent on subcellular localization [18,19]. Considering this unique metabolism of lutein in primates, the non-human primate is the most relevant animal model for investigating lutein’s functional role in the eye and brain. In addition, lutein bioaccumulation patterns during the first few months of age are poorly understood, largely because human infant tissue samples are not readily available. Since lutein’s unique pattern of accumulation can provide clues to its functional role in eyes and brain, we considered it important to describe lutein bioaccumulation patterns in developing rhesus macaques. Therefore, we investigated how bioaccumulation patterns are influenced by levels of carotenoids in infant formulas.

## 2. Materials and Methods

### 2.1. Ethics Statement

All procedures for this specific study were approved by the Institutional Animal Care and Use Committee of Oregon Health and Science University (OHSU IACUC Protocol IS3766) and carried out in accordance with the National Institutes of Health Guide for the Care and Use of Laboratory Animals.

### 2.2. Animals and Diets

Two male and two female rhesus monkeys (*Macaca mulatta*) were group-housed with their dams from birth until 1–3 months of age; the dams were fed Monkey Diet Jumbo 5037 (Lab Diet, St. Louis, MO, USA) supplemented with a variety of fresh fruits and vegetables. The infants were then weaned and formula-fed according to detailed protocols for nursery rearing at ONPRC. One male and one female, starting at 27 and 86 days old, respectively, were fed a formula (Similac^®^ OptiGRO™, Abbott Laboratories, Abbott Park, IL, USA) supplemented with lutein, zeaxanthin, β-carotene, and lycopene, and one male and one female, starting at 63 and 26 days of age, respectively, were fed an unsupplemented formula with low levels of these carotenoids. The carotenoid profiles of the two formulas are provided in Table 1 and the characteristics of the subjects are described in Table 2. Upon weaning, infants were hand-fed if needed until able to self-feed, with feedings initially four times per day for the younger infants and then decreasing to three times per day at 60 days and older. Small amounts of supplemental solid foods low in carotenoids were provided daily to older infants. All infants were fed their respective formula for a duration of four months. The infants were socialized with infants of similar age and housed in cages with age-appropriate blankets, stuffed toys, and other enrichment devices. The infants’ health and behavior was monitored daily by primate veterinarians as well as specialists in primate behavior and psychological well-being. The data reported here are part of a larger study of the effects of lutein supplementation on retinal and brain development.

### 2.3. Plasma, Serum, and Tissue Collection

Fasted 1 mL blood samples were collected in Ethylenediaminetetraacetic acid (EDTA) and processed to obtain plasma at baseline (before the first formula feeding) and after two and four weeks of formula feeding. After four months (133–135 days) of formula feeding, at ages 162 and 221 days for the supplemented group and 160 and 196 days for the unsupplemented group, infant monkeys were humanely euthanized by a veterinary pathologist under deep pentobarbital anesthesia, in accord with the recommendations of the Panel on Euthanasia of the American Veterinary Medical Association.

At the time of death, fasting blood samples were drawn and centrifuged at 800× *g* for 15 min to obtain serum. Tissues for carotenoid analysis were collected rapidly, placed in cryotubes and frozen in liquid nitrogen. From the brain, samples (approximately 0.5–1 g each) were dissected from prefrontal cortex, occipital cortex, superior temporal cortex, striatum, hippocampus and cerebellum. From each retina, 4 mm biopsy punches were used to obtain a macular sample centered on the fovea and samples of the peripheral retina; the vitreous was removed by blotting with filter paper, and the neural retina was gently dissected from the underlying retinal pigment epithelium and choroid.

### 2.4. Carotenoid Analysis

All extractions and analyses were performed under yellow light to prevent light-induced damage of carotenoids. All extracts were stored under argon at −20 °C for less than 48 h before high pressure liquid chromatography (HPLC) analysis.

#### 2.4.1. Formula Extraction for Carotenoids

To 2 mL of formula was added 10 mL 5% KOH (*w*/*v* in methanol) and the mixture was vortexed for 15 s. Five mL of tetrahydrofuran (THF) was added and the mixture was vortexed for 1 min. Ten mL of extraction solvent (dichloromethane/petroleum ether/hexane, 2:4:4) containing 0.005% butylated hydroxytoluene (BHT) was added, vortexed for 1 min, and centrifuged at 800× *g* for 15 min. The upper organic layer was transferred into a test tube. To the bottom layer, 10 mL of extraction solvent was added and the extraction process was repeated one more time. The extract was pooled and evaporated under nitrogen. To the dried extract were added 3 mL deionized water and 3 mL ethanol. This mixture was vortexed for 2 min, sonicated for 3 min and centrifuged at 800× *g* for 5 min at 4 °C. The upper layer was transferred to a 12 × 75 mm test tube and dried under nitrogen in a water bath (40 °C). The dried extract was reconstituted with 50 μL ethanol, vortexed, and sonicated for 30 s. Twenty μL of the reconstituted extract was analyzed by HPLC using a semibore C 30 column [20].

#### 2.4.2. Plasma or Serum Extraction for Carotenoids

Plasma or serum carotenoids were extracted as previously described [21]. Briefly, about 250 μL sample was mixed with an equal volume of ethanol containing 0.1% BHT to precipitate protein and then vortexed for 30 s. One mL hexane was added, vortexed, and centrifuged at 2400 rpm at 4 °C (Centrifuge 5417R, Eppendorf, Hamburg, Germany) for 3 min. The upper hexane layer was removed. The hexane extraction process was repeated two more times and the extract was pooled and evaporated to dryness under argon.

#### 2.4.3. Brain Extraction for Carotenoids

Brain carotenoids were extracted according to the method of Vishwanathan et al. [22]. Brain samples (0.15 g) were homogenized (Power Gen 500, Fisher-Scientific, Hampton, NH, USA) with 0.3 mL of 0.9% NaCl solution and 0.5 mL ethanol. Echinenone (100 ng) dissolved in methanol as an internal standard and 2 mL ethanol were added to the homogenate. The mixture was vortexed vigorously for 2 min and the sides of the tube were scraped down. After incubating at 70 °C for 2 min, 0.5 mL of freshly-prepared 25% sodium ascorbate and 1 mL of 5% NaOH were added. The mixture was saponified in a 60 °C water bath for 20 min. Subsequently, 0.5 mL of distilled water was added and the mixture was placed on ice for 5 min. Five mL of hexane was added, and the mixture was vortexed for 2 min and centrifuged at 1000× *g* at 4 °C (Centrifuge CR3, Jouan, Winchester, VA, USA) for 10 min. The upper hexane phase was removed and reserved. The hexane extraction process was repeated two more times. Hexane was pooled, dried using a Speedvac concentrator (model AS160; Savant, Milford, MA, USA), and evaporated to dryness under argon. Recovery of the internal standard averaged 80%.

#### 2.4.4. Retinal Extraction for Carotenoids

Retina carotenoids were extracted according to the method of Vishwanathan et al. [22]. The retinas were weighed and the tissue was ground with 1 mL 0.85% saline by using a glass rod on ice. To this, 3 mL chloroform-methanol (2:1, *v*/*v*) and 100 ng echinenone (internal standard) in ethanol were added. After vortexing for 30 s, the phases were separated by centrifugation at 800× *g* at 4 °C (Centrifuge CR3, Jouan) for 15 min. The lower chloroform layer was transferred and evaporated to dryness under argon. The extraction was repeated using 3 mL hexane. The hexane layer was combined with the first extract and evaporated to dryness under argon. Recovery of the internal standard averaged 91%.

#### 2.4.5. Adipose Tissue Extraction for Carotenoids

Carotenoids were extracted from adipose tissues according to the method of Sy et al. [23]. Briefly, 125 mg of adipose sample was weighed and homogenized (Power Gen 500, Fisher-Scientific) with 400 μL Phosphate-buffered saline (PBS). Chloroform (500 μL) and 1 mL methanol were added, followed by vortexing for 5 min. After centrifugation at 1200× *g* at 10 °C (Centrifuge CR3, Jouan) for 10 min, the lower phase was collected and evaporated to dryness under argon. Ethanol-KOH (1 mL, 5.5%, *m*/*v*) and 100 μL of freshly prepared ethanol-pyrogallol (1.2%, *m*/*v*) were added to the dry residue and vortexed for 1 min. The mixture was incubated at 37 °C for 90 min for saponification. After incubation, 1 mL distilled water, 100 ng echinenone dissolved in ethanol, and 3 mL hexane were added. Carotenoids were extracted by hexanes using the above-described steps. Recovery of the internal standard was 100%.

#### 2.4.6. Extraction of Other Tissues for Carotenoids

The extraction method used for other tissues has been previously described [24]. Samples were manually homogenized with ethanol containing 0.1% BHT, and 1 mL of saturated KOH solution was added. After saponification in a 60 °C water bath with intermittent vortexing for 30 min, 2 mL of deionized water and 6 mL of hexanes were added. Carotenoids were extracted by hexanes using the above-described steps.

#### 2.4.7. HPLC

**Figure 1:**
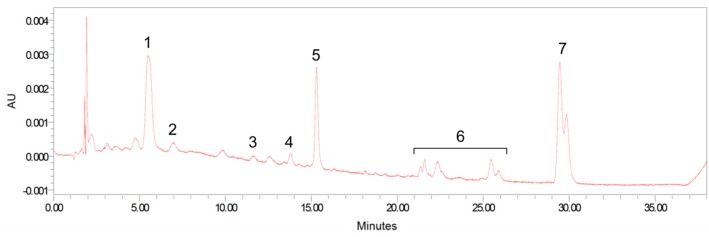
Representative high pressure liquid chromatography (HPLC) separation of carotenoids from serum. Peaks: 1, lutein; 2, zeaxanthin; 3, β-cryptoxanthin; 4, α-carotene; 5, β-carotene; 6, *cis*-lycopene isomers; and 7, all-trans lycopene plus 5-cis lycopene

All analyses were carried out on an Alliance HPLC system (e2695 Separation Module) equipped with 2998 photodiode array detector (Waters, Milford, MA, USA). Sample extracts were reconstituted with 100 μL of ethanol: methyl tert-butyl ether mixture (1:1, *v*/*v*) for brain, retina, and adipose tissue extracts or 40 μL of mobile phase B for serum and the other tissues. The extracts were separated on a reverse-phase C 30 column (4.6 × 150 mm, 3 μm; YMC, Wilmington, NC, USA) maintained at 18 °C. A phase gradient method used for carotenoid separation was based on the method of Yeum et al. [25]. Carotenoids were identified via absorption spectra, retention times, and standard comparison and quantified by an external standard curve method or internal standard curve method. Figure 1 shows a HPLC chromatogram obtained from the serum extract of supplemented formula-fed monkey.

### 2.5. Statistical Analysis

All data were analyzed using SAS software version 9.3 (SAS Institute, Cary, NC, USA). Correlations between two variables were determined by Pearson’s correlation coefficient.

## 3. Results

### 3.1. Formula Carotenoids Profile, Formula Intake, and Body Weight

**Figure 2:**
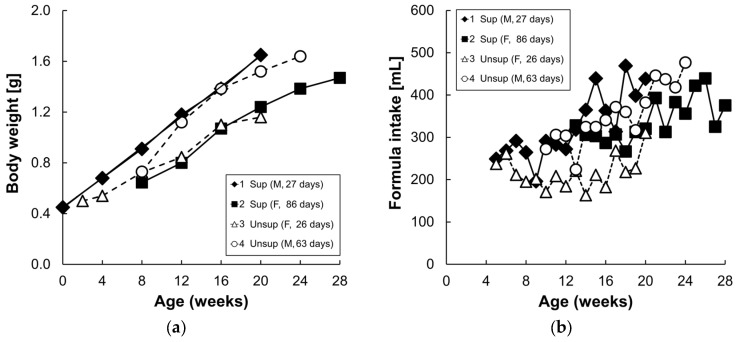
Body weight (**a**) of each infant monkey and daily formula intake consumed by infant monkeys (**b**) for 16 weeks. Each symbol represents one animal; solid symbol and solid line denote supplemented formula (Sup) while the open symbol and dotted line denote unsupplemented formula (Unsup).

The carotenoid profiles of the two formulas are presented in Table 1. The supplemented formula contained higher amounts of lutein and β-carotene (15-fold and three-fold, respectively), compared to the unsupplemented formula. Lycopene and zeaxanthin were present in the supplemented formula but were undetectable in the unsupplemented formula, while α-carotene and β-cryptoxanthin were not detectable in either formula. Formula consumption ranged from 169 to 494 mL/day and increased with age and body weight (Figure 2). There were no consistent differences in formula intake or body weight between the two formula groups.

### 3.2. Plasma/Serum Carotenoids

**Figure 3:**
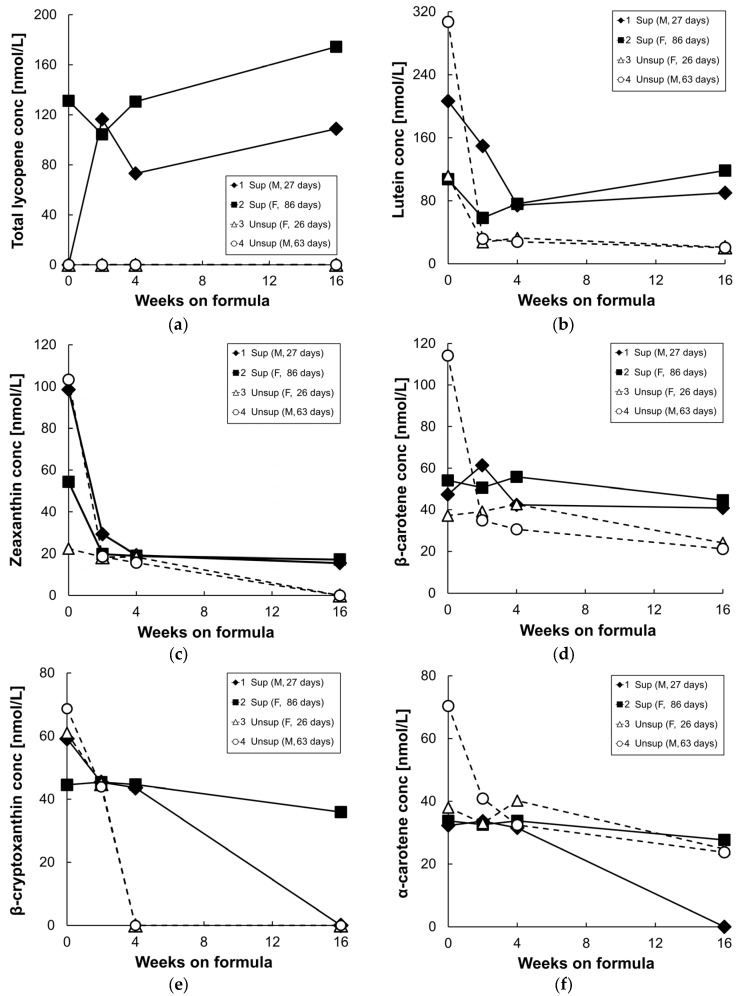
Plasma or serum total lycopene (**a**); lutein (**b**); zeaxanthin (**c**); β-carotene (**d**); β-cryptoxanthin (**e**); and α-carotene (**f**) concentrations of infant monkeys fed either supplemented formula (*n* = 2) or unsupplemented formula (*n* = 2) for 16 weeks. Each symbol represents one animal; solid symbol and solid line denote supplemented formula (Sup) while open symbol and dotted line denote unsupplemented formula (Unsup).

Figure 3 shows plasma/serum carotenoid concentrations at baseline, and following 2, 4, and 16 weeks of formula feeding. At baseline, β-cryptoxanthin, α-carotene, β-carotene, lycopene, lutein, and zeaxanthin were detected in both groups, and the high baseline values are presumably largely due to the highly bioavailable carotenoid supply from breast milk [26]. In addition, the carotenoid levels at baseline were variable, perhaps due to variable breastmilk composition or intake. After 2–16 weeks of formula feeding, levels of all measured carotenoids decreased in the unsupplemented formula group. In the supplemented formula group, only serum lycopene levels increased at 16 weeks compared to their baseline values, due to the high concentration of lycopene in the supplemented formula (Figure 3a). Serum lutein concentrations decreased in both groups from baseline to four weeks on formula, but less so in the supplemented group; as a result, there was a three-fold difference in serum lutein levels between the two groups at 4 weeks (Figure 3b). By 16 weeks on supplemented formula feeding, serum lutein levels increased slightly from levels at four weeks, and were five times higher than in the unsupplemented group. Serum zeaxanthin levels dropped until four weeks of formula feeding in both groups (Figure 3c); they were undetectable in the serum of unsupplemented group by 16 weeks but were maintained in the supplemented group from four to 16 weeks. Serum β-carotene levels also were maintained in the supplemented group from four to 16 weeks and were two times higher than in the unsupplemented group by 16 weeks (Figure 3d). Plasma/serum α-carotene and β-cryptoxanthin levels gradually decreased in both groups from baseline to 16 weeks since these two carotenoids were not present in the formulas (Figure 3e,f).

### 3.3. Brain Carotenoids

**Figure 4:**
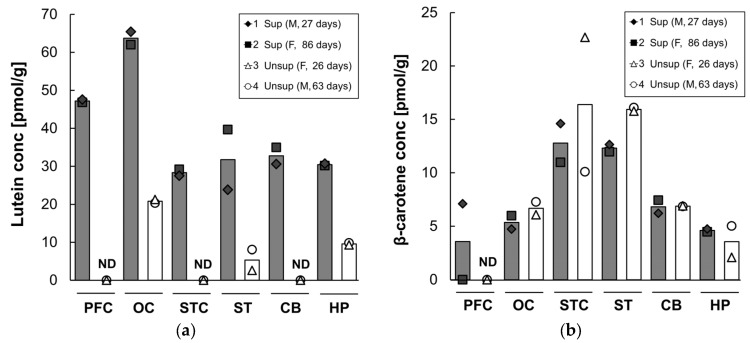
Lutein (**a**) and β-carotene (**b**) concentrations in each brain region of infant monkeys fed either supplemented formula (*n* = 2) or unsupplemented formula (*n* = 2) for 16 weeks. Each symbol represents individual animal; each column represents mean concentration of lutein of two animals. Solid column denotes supplemented formula (Sup) while open column denotes unsupplemented formula (Unsup). ND: not detected; PFC: prefrontal cortex; OC: occipital cortex; STC: superior temporal cortex; ST: striatum; CB: cerebellum; and HP: hippocampus

Lutein and β-carotene concentrations in the prefrontal cortex, occipital cortex, superior temporal cortex, striatum, cerebellum, and hippocampus after 16 weeks on infant formula are shown in Figure 4. Lutein was differentially distributed across the six brain regions, with the highest concentration in the occipital cortex, regardless of the formula type. The supplemented formula resulted in a several-fold increase in lutein deposition in all brain regions compared to the unsupplemented formula. The lutein concentration in the supplemented group was three-fold higher in the occipital cortex and hippocampus (occipital: 64 pmol/g versus 21 pmol/g; hippocampus: 30 pmol/g versus 10 pmol/g) and six-fold higher in the striatum (32 pmol/g versus 5 pmol/g) compared to that of the unsupplemented group. In contrast, in the monkeys fed unsupplemented formula, lutein was undetectable in the prefrontal and superior temporal cortices and cerebellum. Small amounts of β-carotene were detected (range: 0–23 pmol/g) across brain regions. Neither zeaxanthin nor lycopene were detected in any of the brain samples tested in either group. Lutein was the dominant carotenoid in all brain areas regardless of the formula type except for the superior temporal cortex, striatum, and cerebellum of the unsupplemented formula group in which lutein was undetectable or very low.

### 3.4. Retinal Carotenoids

**Figure 5:**
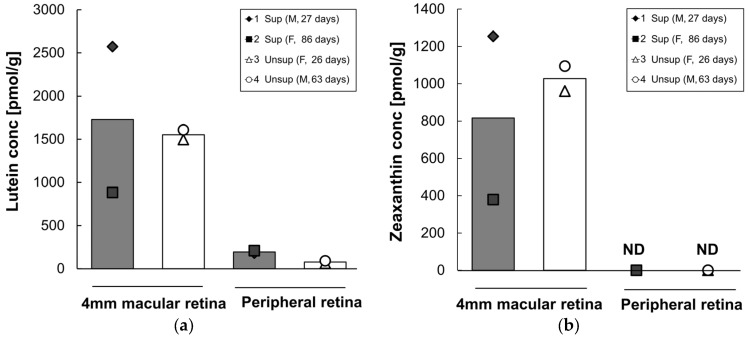
Lutein (**a**) and zeaxanthin (**b**) concentrations in each retina region of infant monkeys fed either supplemented formula (*n* = 2) or unsupplemented formula (*n* = 2) for 16 weeks. Each symbol represents individual animal; each column represents mean concentration of lutein of two animals. Solid column denotes supplemented formula (Sup) while open column denotes unsupplemented formula (Unsup). ND: not detected.

Lutein and zeaxanthin concentrations in each retinal region are presented in Figure 5. As expected, lutein and zeaxanthin were present at high levels in the 4 mm diameter macular retina, whereas lower levels of lutein were present and no detectable zeaxanthin were present in peripheral retina. Mean lutein and zeaxanthin did not differ between formula groups in the 4 mm macular retina. However, in the peripheral retina, the mean lutein content was 2.5 times higher in supplemented monkeys (196 pmol/g) than in the unsupplemented group (71 pmol/g). β-Carotene, lycopene, β-cryptoxanthin, and α-carotene were not detected in any of the retinal regions tested in either group.

Across all four subjects, serum lutein was strongly correlated with peripheral retina lutein (*r* = 0.98, *p* = 0.02), but not with 4 mm macular retina lutein (*r* = −0.09, *p* = 0.91).

### 3.5. Adipose Tissue Carotenoids

Lutein, β-carotene, and lycopene concentrations in mesenteric (MAT), abdominal subcutaneous (ASAT), thigh subcutaneous (TSAT), and axillary brown adipose tissues (BAT) are shown in Table 3. Among the four different adipose areas, ASAT was the highest in lutein, regardless of the formula type. Lutein concentration was increased by supplemented formula in all adipose tissues analyzed, with approximately a three-fold increase in the ASAT (112 pmol/g versus 43 pmol/g), a two-fold increase in the MAT (39 pmol/g versus 17 pmol/g) and TSAT (88 pmol/g versus 39 pmol/g), and a 13-fold increase in BAT (63 pmol/g versus 5 pmol/g). Lycopene was not detectable in any adipose samples from the unsupplemented formula group, but was the predominant carotenoid in adipose tissues in the supplemented group. There were no consistent differences in β-carotene levels in adipose tissues between the groups.

### 3.6. Carotenoids in Other Tissues

Table 4 shows the β-carotene, lutein, zeaxanthin, total lycopene, and α-carotene concentrations in the liver, spleen, lung, kidney, heart, and quadriceps. Lutein was the predominant carotenoid in the liver of both groups. The greatest difference between the formula groups was found in the liver, with the supplemented group showing a nine-fold increase compared with the unsupplemented group, and higher levels in liver than in any of the other organs. Monkeys fed the supplemented formula also had increased lutein concentrations in the spleen, lung, kidney, heart, and quadriceps. However, in contrast to the supplemented group, in the unsupplemented group the lutein concentration of the liver was similar to that of spleen and lung. As in adipose tissues, lycopene was undetectable in the liver, spleen, lung, kidney, heart, and quadriceps from the unsupplemented formula group, but present in all cases in the supplemented group. β-carotene was present in the liver, spleen, lung, kidney, heart, and quadriceps of both formula groups, with somewhat lower levels in the liver, heart and spleen of the unsupplemented group. Zeaxanthin and α-carotene were detectable only in the liver of the supplemented group, while β-cryptoxanthin was undetectable in all tissue samples.

## 4. Discussion

Among dietary carotenoids, lutein is preferentially deposited in the human infant brain and retina, a finding that suggests a role in the development of these tissues [13,22]. Although lutein has been supplemented in some commercial infant formulas, its bioaccumulation pattern is not well understood in early life stages. This pilot study is the first report to describe lutein bioaccumulation patterns in nonhuman primate infants fed infant formulas with high or low levels of carotenoids.

Interest in the potential role of lutein in brain development has been prompted by studies showing that this carotenoid is selectively accumulated in the brain of infants, as well as adults [6,7]. A primary finding of the current study was that four months of feeding a carotenoid supplemented formula led to higher concentrations of lutein in all brain regions tested. We also determined that lutein accumulated differentially across brain areas. Lutein accumulation was highest in the occipital cortex, the primary visual processing area, which is consistent with an earlier report on brain carotenoids in xanthophyll-free adult monkeys fed pure lutein [22]. In contrast, in human tissue, the highest lutein accumulation was found in the cerebellum of centenarians [6] and in the auditory cortex of infants [7]. This suggests that lutein’s accumulation across brain regions might differ between species and/or across the lifespan. While serum levels and ratios of carotenoids largely reflected their levels in the two formulas, in the infant brain the supplemented formula resulted in a specific and selective increase in lutein, with only small amounts of β-carotene and undetectable levels of the other carotenoids despite enhanced intake. Notably, we found that lutein was undetectable in prefrontal and superior temporal cortices and cerebellum of the unsupplemented formula group. This might suggest differences in the turnover rate of lutein across brain regions. Yonekura et al. showed different half-lives of lutein metabolites in multiple tissues of male mice as follows: plasma < liver < kidney << adipose tissues [27].

It is interesting to note that lutein concentration in the lutein-supplemented group was increased in the peripheral retina, but not in the 4 mm macular retina. We also observed a correlation between serum lutein and peripheral retina lutein, but not between serum lutein and macular retina lutein. One possible explanation is that lutein was preferentially deposited in the macular region in the first few weeks of life, prior to the initiation of formula feeding, whereas lutein in the peripheral retina continued to increase over the next four months in the supplemented group. Johnson et al. previously showed that lutein in both foveal and peripheral retina was increased by a high level of lutein supplementation in adult monkeys initially devoid of xanthophylls [28]. Many human clinical studies show that 2.4–30 mg of daily lutein supplementation enhances macular xanthophyll levels, as measured by MPOD, in healthy adults and in subjects with retinal diseases [29,30,31,32,33]. Johnson et al. also showed that lutein supplementation increased MPOD at several eccentricities in older women [34]. However, little is known about differential lutein deposition in the macular and peripheral retina, particularly in early life. These findings might be an important consideration when measuring MPOD in children, as several methods for measuring MPOD are based on comparing foveal to more peripheral retinal locations with regard to the relative absorption of short wavelength blue light, strongly absorbed by lutein and zeaxanthin, to middle wavelength light that is minimally absorbed [35].

Interestingly, we observed that lycopene, a non-hydroxylated carotene transported mainly by low-density lipoprotein (LDL) [36,37], was not found in any brain or retinal region. This lack of brain lycopene agrees with findings for the brains of human infants [7], whereas small amounts of lycopene (four-fold less than lutein) were found in the brain of centenarians [6]. As shown in Table 3 and Table 4, lycopene is readily taken up in body tissues of infant monkeys. Lutein, a polar hydroxylated carotenoid, is primarily transported by high-density lipoprotein (HDL) and to a lesser extent by LDL in the circulation [38]. In the retina, lutein is taken up via a scavenger receptor class B type I (SR-BI)-dependent mechanism [39], and protected by binding to a specific lutein binding protein, steroidogenic acute regulatory domain protein 3 (StARD3) [40]. Recent findings showed that lutein concentrations are related to levels of this protein in human brain [41], and that the relationship is particularly strong in the pediatric brain. Further study is required to determine the mechanism underlying preferential uptake of lutein, which may involve both HDL transport and StARD3.

It is also noteworthy that lutein’s distribution is not homogenous among adipose depots. This result parallels a previous study showing site-specific lutein distribution in the subcutaneous adipose tissues in healthy human subjects [42]. One possible explanation for this regional lutein distribution is HDL metabolism. Transport by HDL may enhance lutein uptake into tissues. Despres et al. showed in obese patients that more HDL was taken up by subcutaneous adipocytes than omental adipocytes [43]. This difference may explain the higher lutein accumulation in subcutaneous adipose compared to visceral adipose. Furthermore, cholesteryl ester transfer protein is known to mediate selective uptake of HDL-cholesteryl ester in human adipocytes, and its mRNA expression is up-regulated in subcutaneous fat compared to visceral fat [44,45].

Lutein has both antioxidant and anti-inflammatory effects in vitro and in vivo. Considering that infants can be at special risk of oxidative stress, lutein’s role as an antioxidant may be essential in early life. In an in vitro model of LPS-stimulated macrophages, lutein scavenged H_2_O_2_ and superoxide anions, decreasing the level of intracellular H_2_O_2_ accumulation and thereby inhibiting the NF-κB pathway [46]. Similarly, lutein treatment reduced the expression of inflammatory molecules by suppressing NF-κB activation in three types of cells associated with choroidal neovascularization [47]. Supplemental lutein prevented hypercholesterolemic diet-induced atherosclerosis in guinea pigs by decreasing malondialdehyde and oxidized-LDL [48]. In addition, lutein administration in the first hours of life increased biological antioxidant potential and decreased hydroperoxides compared to untreated human newborns who were exposed to oxidative stress [15]. Collectively, these earlier findings support the hypothesis that dietary lutein has a protective effect for many organs during early life.

## 5. Conclusions

In conclusion, in our small pilot study we found that increased early exposure to dietary lutein leads to enhancement of lutein tissue deposition. Notably, we found differential deposition of lutein in brain and adipose areas. Additional studies will be required to determine if lutein has a different half-life across different brain areas and other tissues and, more importantly, if lutein has a functional role in development of the brain, retina, and other organ systems.

## Figures and Tables

**Table 1:** Carotenoid profiles of the infant formulas ^1^.

Formula Type	Lutein	Zeaxanthin	β-Carotene	Lycopene	β-Cryptoxanthin	α-Carotene
Supplemented formula (Similac)	248	23	88	362	ND ^2^	ND
Unsupplemented formula	16	ND	32	ND	ND	ND

^1^ Data are expressed in nmol/L; ^2^ ND, not detected.

**Table 2:** Monkey characteristics.

Group, Animal ID	Gender	Age (Days) at Enrollment
Supplemented formula		
1	Male	27
2	Female	86
Unsupplemented formula		
3	Female	26
4	Male	63

**Table 3:** β-carotene, lutein, and total lycopene concentrations in each adipose region.

Carotenoid	Group, Animal ID	MAT	ASAT	TSAT	BAT
		Mean (Individual Values), pmol/g			
β-carotene	Supplemented	77	125	56	105
	(1, 2)	(49, 105)	(91, 159)	(68, 45)	(107, 102)
	Unsupplemented	82	83	100	59
	(3, 4)	(61, 103)	(32, 133)	(131, 68)	(43, 75)
Lutein	Supplemented	39	112	88	63
	(1, 2)	(25, 53)	(71, 154)	(69, 107)	(78, 47)
	Unsupplemented	17	43	39	5
	(3, 4)	(13, 21)	(35, 52)	(34, 43)	(ND, 10)
Total lycopene	Supplemented	227	391	350	333
	(1, 2)	(162, 291)	(280, 502)	(220, 481)	(295, 371)
	Unsupplemented	ND ^1^	ND	ND	ND
	(3, 4)				

MAT: mesenteric adipose tissue; ASAT: abdominal subcutaneous adipose tissue; TSAT: thigh subcutaneous adipose tissue; BAT: brown adipose tissue. ^1^ ND: not detected.

**Table 4:** β-carotene, lutein, zeaxanthin, total lycopene, and α-carotene, concentrations in liver, lung, kidney, heart, quadriceps, and spleen.

Carotenoid	Group (Animal ID)	Liver	Lung	Kidney	Heart	Quadriceps	Spleen
		Mean (Individual Values), pmol/g					
β-carotene	Supplemented	56	134	118	122	116	105
	(1, 2)	(51, 61)	(125, 142)	(121, 115)	(124, 120)	(117, 115)	(86, 125)
	Unsupplemented	29	128	113	57	111	59
	(3, 4)	(29, 30)	(131, 125)	(103, 122)	(0, 114)	(117, 104)	(56, 63)
Lutein	Supplemented	505	120	67	60	54	227
	(1, 2)	(453, 557)	(129, 111)	(66, 67)	(63, 56)	(52, 56)	(219, 236)
	Unsupplemented	59	69	35	35	34	67
	(3, 4)	(54, 63)	(93, 44)	(30, 39)	(34, 35)	(37, 31)	(71, 63)
Zeaxanthin	Supplemented	34	ND^1^	ND	ND	ND	ND
	(1, 2)	(33, 35)					
	Unsupplemented	ND	ND	ND	ND	ND	ND
	(3, 4)						
Total lycopene	Supplemented	376	209	183	148	150	278
	(1, 2)	(253, 500)	(174, 243)	(167, 198)	(134, 161)	(105, 194)	(139, 418)
	Unsupplemented	ND	ND	ND	ND	ND	ND
	(3, 4)						
α-carotene	Supplemented	18	ND	ND	ND	ND	ND
	(1, 2)	(ND, 35)					
	Unsupplemented	34	ND	ND	ND	ND	ND
	(3, 4)	(33, 35)					

^1^ ND: not detected.
